# Announcement: 5th International Conference on Environmental and Biological Aspects of Main-Group Organometals (ICEBAMO 2001)

**DOI:** 10.1155/MBD.2000.289

**Published:** 2000

**Authors:** Walter Goessler

**Affiliations:** Institute of Chemistry Analytical Chemistry KarI-Franzens-University Universitätsplatz 1 Graz A-8010 Austria


					Metal Based Drugs Vol. 7, Nr. 5, 2000
ICEBAMO 2001
5th INTERNATIONAL CONFERENCE ON ENVIRONMENTAL AND
BIOLOGICAL ASPECTS OF MAIN-GROUP ORGANOMETALS
05.06.2001 09.06.2001
Castle of Schielleiten, Styria, Austria
organized by the
Institute of Chemistry Analytical Chemistry
KarI-Franzens-University Graz, Austria
http://www.kfunigraz.ac.at/achwww/icebamo
Goals and Scope
The ICEBAMO meetings aim to facilitate interdisciplinary discussion on environmental and
biological aspects of main-group organometals. Previous meetings in Padua (1986 and 1991),
Bordeaux (1994), and Odense (1998) have brought together researchers from the fields of
toxicology, environmental sciences, analytical chemistry, biochemistry, and synthetic
organometallic chemistry. ICEBAMO 2001 at the castle of Schieileiten, which is located about 50
km east of Graz, Styria, Austria, will provide a forum for discussing the origin, behavior and fate of
organometallic compounds in the environment, and their application to and impact on biological
systems.
Focus will be on the following aspects of organometallic compounds:
Origin and pathways in the environment
Fate in organisms including accumulation and toxicological effects
Biological and biochemical studies on transformation/detoxification processes
Analytical chemistry including speciation techniques
Main group metal-based drugs
The meeting will consist of oral presentations of invited and contributed papers and poster
presentations.
Invited oral presentations will be 45 minutes.
Contributed oral presentations will be 20 minutes including 5 minutes for questions.
Posters will be an important part of the conference. Time will be available each day for viewing.
Venue
The baroque castle of Schielleiten is located about 50 km northeast of Graz, the capital of Styria. In
addition to the ideal background for a conference, a wide range of different sports facilities (e.g.
football, tennis, fitness studio) is offered. The nearby lake Stubenbergsee inivites to all kinds of
water sports. Schielleiten is reached from Graz via the routes B74 or A2 and B54.
Bus transfer from Graz (airport, railway station) to Schielleiten will be offered.
289
ICEBAMO 2001
Contact Address"
Analytical Chemistry
Walter Goessler
Institute of Chemistry
KarI-Franzens-University
A-8010 Graz, Austria
Universititsplatz 1
Phone: 0043 316 380 5302 Fax- 0043 316 380
<waIter.goess er~kfunigraz.ac.at>
9845
290

				

